# Cannabidiol Alleviates Aβ_1–42_-Induced Cognitive Deficits and Neuronal Injury Associated with Modulation of the AEA–FAAH–CB1R Axis and BDNF/TrkB Signaling

**DOI:** 10.3390/biom16101473

**Published:** 2026-10-10

**Authors:** Yan Kong, Juan Peng, Jian Sun, Yinglan Nie

**Affiliations:** 1 Experimental Research Center, China Academy of Chinese Medical Sciences, Beijing 100700, China; y17860506421@163.com (Y.K.); pengjuan@merc.ac.cn (J.P.); sunjian@merc.ac.cn (J.S.); 2 Institute of Chinese Materia Medica, China Academy of Chinese Medical Sciences, Beijing 100700, China

**Keywords:** Alzheimer’s disease, cannabidiol, Aβ_1–42_, endocannabinoid system, CB1 receptor, BDNF/TrkB signaling, neuroprotection

## Abstract

**Background:** Alzheimer’s disease (AD) is a progressive neurodegenerative disorder characterized by cognitive decline, synaptic dysfunction, and neuronal injury. Cannabidiol (CBD), a non-psychoactive constituent of Cannabis sativa L., has neuroprotective potential, but its underlying mechanisms remain unclear. **Purpose:** This study investigated the effects of CBD on Aβ_1–42_-induced cognitive impairment and hippocampal injury, focusing on endocannabinoid and BDNF/TrkB signaling. **Methods:** Cognitive function was assessed by Morris water maze and novel object recognition tests; hippocampal pathology and molecular changes were evaluated by H&E staining, immunofluorescence, LC–MS, ELISA, and Western blotting; and CB1R involvement was further examined in Aβ_1–42_-treated PC12 cells using AM251. **Results:** CBD improved cognitive performance, attenuated hippocampal histopathological alterations, reduced tau hyperphosphorylation, and partially restored synaptophysin levels. CBD also increased hippocampal AEA levels, reduced FAAH levels, and restored CB1R and BDNF/TrkB-associated signaling. In Aβ_1–42_-treated PC12 cells, CBD improved cell viability and increased synaptophysin, CB1R, BDNF, and p-TrkB/TrkB levels, whereas AM251 attenuated several CBD-associated effects. **Conclusions:** These findings suggest that modulation of the AEA–FAAH–CB1R axis and BDNF/TrkB signaling may contribute to the neuroprotective effects of CBD against Aβ_1–42_-induced cognitive impairment and neuronal injury.

## 1. Introduction

Alzheimer’s disease (AD) is the leading cause of dementia and a progressive neurodegenerative disorder that profoundly affects cognitive function and quality of life in older adults [1]. Clinically, AD is characterized by progressive memory impairment, cognitive decline, and loss of daily living abilities. Pathologically, AD is associated with amyloid-β (Aβ) accumulation, tau hyperphosphorylation, synaptic dysfunction, neuroinflammation, and neuronal loss [2,3]. An estimated 57 million people worldwide are living with dementia, and this number is projected to reach 139 million by 2050 [4], with AD accounting for 60–70% of cases [5,6]. Current symptomatic treatments for AD include acetylcholinesterase inhibitors such as donepezil, which can provide beneficial effects on cognitive function [7]. However, these treatments do not halt or reverse disease progression. Thus, identifying safe and effective neuroprotective candidates that can attenuate Aβ-related neuronal injury and cognitive impairment remains an important goal in AD research.

Among emerging neuroprotective candidates, cannabidiol (CBD), a major non-intoxicating constituent of *Cannabis sativa* L., has attracted increasing interest as a potential neuroprotective compound because of its antioxidant, anti-inflammatory, and anti-apoptotic properties [8,9,10]. Accumulating preclinical evidence indicates that CBD may modulate several pathological processes relevant to AD, including neuroinflammation, oxidative stress, synaptic impairment, and Aβ-induced neuronal injury [11,12,13]. These effects may be linked, at least in part, to the endocannabinoid system (ECS), which is involved in the regulation of neurotransmission, inflammatory responses, synaptic plasticity, and cognitive function [14,15].

Anandamide (AEA) is a major endocannabinoid that is primarily degraded by fatty acid amide hydrolase (FAAH) [16,17]. By reducing FAAH-mediated AEA degradation, CBD may enhance endocannabinoid tone and modulate cannabinoid receptor-related signaling [18]. Among cannabinoid receptors, cannabinoid type 1 receptor (CB1R) is highly expressed in the central nervous system and is involved in synaptic transmission, neuronal excitability, and memory-related processes [19,20]. Emerging evidence also indicates functional crosstalk between CB1R and brain-derived neurotrophic factor (BDNF)/tropomyosin receptor kinase B (TrkB) signaling [21,22,23]. BDNF/TrkB signaling is a key regulator of neuronal survival, synaptic plasticity, and functional recovery after neural injury [24,25,26]. Physiologically, BDNF binding to TrkB promotes long-term potentiation and other forms of activity-dependent synaptic plasticity that are essential for the stabilization and consolidation of newly acquired memories [27]. BDNF/TrkB signaling also contributes to synaptic structural integrity by supporting the formation, maturation, and maintenance of dendritic spines and synaptic connections [28]. Accordingly, impairment of BDNF/TrkB signaling may compromise synaptic stability and memory consolidation, thereby contributing to cognitive dysfunction under AD-related pathological conditions. These observations suggest that the AEA–FAAH–CB1R axis may interact with BDNF/TrkB signaling to regulate synaptic and neuronal responses under AD-related pathological conditions.

However, whether CBD protects against Aβ_1–42_-induced cognitive impairment and neuronal injury through coordinated regulation of the ECS and BDNF/TrkB signaling remains unclear. In particular, the contribution of CB1R to CBD-associated modulation of BDNF/TrkB signaling under Aβ-induced injury conditions has not been fully defined. In the present study, we used Aβ_1–42_-induced AD model mice to evaluate the effects of different doses of CBD on learning and memory, hippocampal injury, tau phosphorylation, synaptic protein abundance, and ECS-related markers. We further used Aβ_1–42_-treated PC12 cells, together with the CB1R antagonist AM251, to investigate whether CB1R participates in CBD-mediated regulation of BDNF/TrkB signaling.

## 2. Materials and Methods

### 2.1. Materials and Reagents

Cannabidiol powder (CBD; purity, 99.9%; batch no. CP0120190501) was obtained from Heilongjiang Zhongsheng Biotechnology Co., Ltd. (Daqing, China). Donepezil tablets (batch no. 14202012042) were purchased from Eisai China Inc. (Suzhou, China). Aβ_1–42_ (E1375) and dimethyl sulfoxide (DMSO; D2650) were purchased from Sigma-Aldrich (St. Louis, MO, USA). PC12 cells were obtained from the Cell Resource Center, Peking Union Medical College, Chinese Academy of Medical Sciences (Beijing, China). HRP-conjugated goat anti-rabbit IgG antibody (ab205718) and HRP-conjugated goat anti-mouse IgG antibody (ab6789) were purchased from Abcam (Cambridge, UK). Anti-Tau antibody (346514) and anti-p-Tau antibody (381213) were obtained from Zhengneng Biotechnology Co., Ltd. (Chengdu, China). Anti-BDNF antibody (BD029795), Anti-TrkB antibody (BC031835), Anti-CB1R antibody (BC074812), anti-synaptophysin antibody (BC064550), anti-rabbit IgG-HRP antibody (SA01002), anti-GAPDH antibody (SH0007007), and anti-β-actin antibody (BC002409) were purchased from Wuhan Sanying Biotechnology Co., Ltd. (Wuhan, China). Anti-phospho-TrkB (Tyr515) antibody (386C8A10) was purchased from Thermo Fisher Scientific (Waltham, MA, USA).

### 2.2. Animals and Drug Administration

A total of 84 male ICR mice, aged 4–6 weeks and weighing 18–22 g, were purchased from Beijing Huafukang Bio-Technology Co., Ltd. (Beijing, China). All animals were maintained under specific pathogen-free (SPF) conditions and allowed free access to food and water. After 1 week of acclimatization, mice were randomly assigned to seven groups (*n* = 12 per group): control, model, donepezil, low-dose CBD (CBD-L), medium-dose CBD (CBD-M), high-dose CBD (CBD-H), and sham-operated groups.

Oral administration was initiated on the day after stereotaxic injection. The CBD-L, CBD-M, and CBD-H groups received CBD by oral gavage at 28, 70, and 140 mg/kg/day, respectively. The donepezil group received donepezil by oral gavage at 1.3 mg/kg/day. Donepezil, a clinically established acetylcholinesterase inhibitor used for the symptomatic treatment of AD, was included as a positive-control treatment to provide a clinically relevant reference for evaluating the effects of CBD. The doses of CBD and donepezil were selected based on conversion from clinically relevant equivalent doses. The control, sham-operated, and model groups received an equivalent volume of vehicle by oral gavage (0.2 mL/10 g body weight).

All treatments were administered once daily. Mice received treatment for 21 consecutive days before the initiation of behavioral testing. The CBD doses of 28, 70, and 140 mg/kg/day were designated as low, medium, and high dose levels, respectively, to evaluate the effects of CBD across a dose range. The 21-day pre-behavioral treatment period was selected based on preliminary experiments conducted by our research group, in which this regimen produced a relatively favorable and stable therapeutic response.

After the initial 21-day treatment period, the MWM test was conducted over 6 consecutive days, followed by the NOR test over 3 consecutive days. Daily administration of CBD, donepezil, or vehicle was continued throughout the behavioral testing period, and the MWM and NOR tests were conducted approximately 1 h after each daily oral administration. Following completion of all behavioral assessments, the mice received the final administration, and tissue samples were collected approximately 1 h later. The overall experimental design and timeline are illustrated in Figure 1.

Male ICR mice were acclimatized for 1 week before model induction. Aβ_1–42_ was administered by intracerebroventricular (i.c.v.) injection to establish the experimental model, whereas the sham-operated group received an equal volume of saline. Oral administration of CBD (28, 70, or 140 mg/kg/day), donepezil (1.3 mg/kg/day), or vehicle was initiated on the day after stereotaxic injection and performed once daily. After 21 consecutive days of treatment, mice underwent the Morris water maze (MWM) test for 6 consecutive days, followed by the novel object recognition (NOR) test for 3 consecutive days. Daily treatment was continued throughout behavioral testing, and behavioral assessments were conducted approximately 1 h after daily oral administration. Following completion of all behavioral assessments, the mice received the final administration, and tissue samples were collected approximately 1 h later.

### 2.3. Aβ_1–42_ Preparation

Aβ_1–42_ peptide was dissolved in hexafluoroisopropanol (HFIP) at 4.5 mg/mL and incubated at room temperature for 1 h. HFIP was then allowed to evaporate completely to form a peptide film. The film was reconstituted in dimethyl sulfoxide (DMSO) at 22.5 mg/mL and sonicated for 10 min. Phosphate-buffered saline (PBS) was then added to dilute the Aβ_1–42_ solution to 2.5 mg/mL, followed by incubation at 4 °C for 24 h. The preparation was further diluted with PBS to a final concentration of 1 mg/mL and incubated at 4 °C for 2 weeks. The resulting pre-incubated Aβ_1–42_ preparation, generated under aggregation-promoting conditions, was used for subsequent experiments.

### 2.4. Stereotaxic Injection and AD Model Mice Establishment

The AD model was established by bilateral intracerebroventricular injection of Aβ_1–42_. Mice were anesthetized with isoflurane, and anesthesia was maintained throughout the stereotaxic procedure. The mice were then secured in a stereotaxic apparatus. Injection coordinates were determined according to a mouse brain stereotaxic atlas as follows: anteroposterior, −0.34 mm from bregma; mediolateral, ±1.0 mm; and dorsoventral, −2.0 mm. Aβ_1–42_ (1 μL, 1 mg/mL) was injected into each lateral ventricle. Mice in the control group received no injection, whereas mice in the sham-operated group received an equal volume of saline. Following stereotaxic surgery, gentamicin sulfate was administered intramuscularly at 5 mg/kg once daily for 3 consecutive days for postoperative infection prophylaxis.

### 2.5. Morris Water Maze (MWM) Test

During the acquisition phase, conducted on days 1–6, the maze was divided into four quadrants, and the hidden platform was placed in quadrant II, 2 cm below the water surface. Skim milk powder was added to the water to obscure the platform. Each mouse was given 60 s to locate the hidden platform, and the time required to reach the platform was recorded as the escape latency.

On day 6, after completion of the final acquisition trial, a probe trial was performed. The platform was removed, and each mouse was released into the water from the quadrant opposite the original platform location. During the 60 s trial, the number of platform crossings, time spent in the target area, swimming distance, and swimming speed were recorded.

### 2.6. Novel Object Recognition (NOR) Test

The NOR test was used to evaluate cognitive function based on the innate preference of mice for novel objects and their ability to discriminate between familiar and novel objects. Mice were habituated to the experimental environment in two 10 min sessions. Two identical square objects were then placed diagonally in the test box, and mice were allowed to explore freely for 10 min.

During the test session, one familiar square object was retained, whereas the other was replaced with a novel round object placed diagonally in the box. Each mouse was allowed to explore for 5 min. The time spent exploring the familiar and novel objects was recorded. The discrimination index (DI), defined as the proportion of total object exploration time spent exploring the novel object, was calculated as follows:

DI = time spent exploring the novel object/(time spent exploring the novel object + time spent exploring the familiar object).

### 2.7. Tissue Collection

Following completion of all behavioral assessments, the mice received the final drug administration, and tissue samples were collected approximately 1 h later. For histological analyses, three mice from each group were deeply anesthetized with isoflurane and subjected to transcardial perfusion. The brains were then removed and fixed in 4% paraformaldehyde for H&E staining and immunofluorescence analysis. The remaining mice used for biochemical analyses were deeply anesthetized and euthanized by isoflurane overdose. The brains were rapidly removed, and the hippocampi were dissected on ice and stored at −80 °C until further biochemical analyses.

### 2.8. H&E Staining

Brain tissue was fixed in 4% paraformaldehyde, dehydrated in increasing concentrations of alcohol, and embedded in paraffin. Paraffin-embedded tissues were sectioned at a thickness of 4 μm. Sections were mounted on glass slides, dried, baked, and deparaffinized in xylene I and II for 20 min each. The sections were then rehydrated in absolute ethanol I and II for 10 min each, followed by 95%, 90%, 80%, and 70% ethanol for 5 min each. After hematoxylin and eosin staining, the sections were dehydrated, cleared, coverslipped, and examined under a light microscope as previously described [29].

### 2.9. Immunofluorescence

Sections were deparaffinized, subjected to antigen retrieval, and blocked with 10% goat serum at 37 °C for 30 min. For sequential TSA-based double immunofluorescence staining, sections were first incubated with anti-Tau antibody (346514; 1:200) at 4 °C overnight, followed by the corresponding HRP-conjugated secondary antibody at 37 °C for 1 h and Cy3 tyramide (1:1000) at 37 °C for 30 min. After a second antigen-retrieval step and serum blocking, sections were incubated once with anti-p-Tau antibody (381213; 1:200) at 4 °C overnight, followed by the corresponding HRP-conjugated secondary antibody at 37 °C for 1 h and Alexa Fluor 488 tyramide (1:1000) at 37 °C for 30 min. The HRP-conjugated secondary antibodies used were goat anti-rabbit IgG (ab205718; 1:2000) and goat anti-mouse IgG (ab6789; 1:500). Nuclei were counterstained with DAPI for 5 min at room temperature, and sections were mounted with antifade mounting medium.

### 2.10. LC–MS Analysis of Hippocampal AEA and 2-AG

#### 2.10.1. Sample Preparation

Hippocampal tissue was accurately weighed and homogenized with ultrapure water at a tissue-to-water ratio of 1:5 (*w*/*v*). The homogenate was centrifuged at 6200× *g* and 4 °C for 10 min, and the supernatant was discarded. Methanol was added to the pellet at a ratio of 1:4, followed by vortex mixing to extract AEA and 2-AG. Samples were centrifuged again at 19,100× *g* and 4 °C for 10 min, and the resulting supernatant was collected for analysis.

#### 2.10.2. Chromatographic Conditions

Chromatographic separation was performed using a Waters ACQUITY UPLC HSS T3 column (1.8 μm, 2.1 × 100 mm; Waters). The mobile phase consisted of 10 mM ammonium acetate (A) and acetonitrile (B). Isocratic elution was performed at a flow rate of 0.3 mL/min. The injection volume was 1 μL, and the column temperature was maintained at 30 °C.

#### 2.10.3. Mass Spectrometric Conditions

Mass spectrometric detection was performed using an electrospray ionization (ESI) source operated in positive ion mode with multiple reaction monitoring (MRM). The monitored ion transitions were *m*/*z* 348.3 → 62.3 for AEA and *m*/*z* 379.5 → 287.3 for 2-AG. The collision energy was set at 10 V, and the fragmentor voltage was set at 135 V. The nebulizer gas pressure was 15 psi, the ion source temperature was 300 °C, and the nebulizer gas flow rate was 12 L/min.

### 2.11. Enzyme-Linked Immunosorbent Assay (ELISA)

FAAH levels in hippocampal samples were determined using a commercially available FAAH ELISA kit (Jianglai Biotechnology, Shanghai, China; Cat. No. 202307) according to the manufacturer’s instructions. Standard, blank, and sample wells were prepared as specified by the kit. For each sample well, 40 μL of sample diluent and 10 μL of sample were added, corresponding to a five-fold dilution. The plate was sealed and incubated at 37 °C for 1 h. After incubation, the liquid was discarded, and each well was washed five times with washing buffer, with the washing solution allowed to remain in the wells for approximately 30 s during each wash. Subsequently, 50 μL of HRP-conjugated detection reagent was added to each well except the blank wells, followed by incubation at 37 °C for 30 min. The wells were then washed five additional times. Next, 50 μL each of chromogenic solutions A and B were added to each well, and the plate was incubated at 37 °C in the dark for 15 min. The reaction was terminated by adding 50 μL of stop solution to each well. Absorbance was measured at 450 nm within 15 min using a Synergy™ microplate reader (BioTek Instruments, Inc., Winooski, VT, USA). FAAH concentrations were calculated from the corresponding standard curve.

### 2.12. Western Blot

Hippocampal tissues were homogenized on ice in RIPA lysis buffer containing protease and phosphatase inhibitors. The lysates were centrifuged at 12,000× *g* for 15 min at 4 °C, and protein concentrations in the supernatants were determined using a BCA assay. Equal amounts of protein (25 μg per lane) were separated by 10% SDS-PAGE and transferred onto PVDF membranes by wet transfer at 300 mA for 50 min. The membranes were blocked with 5% BSA for 1 h at room temperature and incubated overnight at 4 °C with the following primary antibodies: p-TrkB (1:1500), TrkB (1:3500), BDNF (1:2500), CB1R (1:2500), synaptophysin (1:4000), GAPDH (1:5000), and β-actin (1:3000). After washing three times with TBST for 10 min each, the membranes were incubated with HRP-conjugated goat anti-rabbit IgG secondary antibody (1:6000) for 1 h at room temperature. Protein bands were visualized using an ECL substrate (Meilunbio, China) and captured using a 680RGB chemiluminescence imaging system (GE, USA). Band intensities were quantified using ImageJ version 1.54f. Synaptophysin was normalized to GAPDH, CB1R and BDNF to β-actin, and p-TrkB to total TrkB.

### 2.13. Cell Experiments

#### 2.13.1. Cell Culture

Rat adrenal pheochromocytoma PC12 cells were obtained from the Cell Resource Center, Peking Union Medical College, Chinese Academy of Medical Sciences. Frozen cells were rapidly thawed in a 37 °C water bath, supplemented with fresh complete culture medium consisting of high-glucose DMEM containing 10% FBS and 1% antibiotics, and centrifuged at 300× *g* for 5 min. After centrifugation, the supernatant was discarded, and the cell pellet was resuspended in fresh complete medium. Cells were gently pipetted to ensure uniform dispersion, transferred to culture flasks, and maintained at 37 °C in a humidified incubator containing 5% CO_2_.

#### 2.13.2. Effects of CBD and Aβ_1–42_ on PC12 Cell Viability

The effects of CBD and Aβ_1–42_ on PC12 cell viability were evaluated using the Cell Counting Kit-8 (CCK-8) assay. Results were expressed as the percentage of viable cells.

PC12 cells in the logarithmic growth phase, at approximately 70–80% confluence, were seeded into 96-well plates at a density of 8 × 10^4^ cells/mL and cultured for 24 h. Cells were then assigned to the following groups: blank control, CBD treatment, and Aβ_1–42_ treatment groups. The CBD treatment groups were exposed to complete DMEM containing CBD at final concentrations of 2.5, 5, 10, 15, 20, 25, or 30 μM. The Aβ_1–42_ treatment groups were exposed to complete DMEM containing Aβ_1–42_ at final concentrations of 0.5, 1, 2.5, 5, 10, or 20 μM. The blank control group received complete medium alone. Each group included three replicate wells, and cells were incubated for an additional 24 h.

After treatment, the culture medium was removed, and cells were washed three times with PBS. A 10% CCK-8 solution was added to each well, and plates were incubated at 37 °C for 1.5 h. The optical density (OD) was measured at 450 nm using a microplate reader. Cell viability was calculated according to the following formula:

Cell viability (%) = (A_experimental_ − A_blank_)/(A_control_ − A_blank_) × 100%

A_experimental_, A_blank_, and A_control_ represent the absorbance values of the treated, blank, and control wells, respectively.

#### 2.13.3. Cell Treatment and Experimental Grouping

PC12 cells in the logarithmic growth phase were used for all in vitro experiments. When the cells reached approximately 75% confluence, they were allocated to the following groups: control, CBD control, model, CBD treatment, and AM251 treatment groups. Cells in the control group were left untreated. Cells in the CBD control group were exposed to 10 μM CBD for 24 h. Cells in the model group were treated with 1 μM Aβ_1–42_ for 24 h. Cells in the CBD treatment group were co-treated with 1 μM Aβ_1–42_ and 10 μM CBD for 24 h. Cells in the AM251 treatment group were pretreated with 1 μM AM251 for 30 min before exposure to 1 μM Aβ_1–42_ and 10 μM CBD for 24 h.

#### 2.13.4. Light Microscopic Assessment of Cell Morphology

After 24 h of treatment, cell morphology was examined using a light microscope. Representative images were acquired at 20× magnification.

### 2.14. Statistical Analysis

Statistical analyses were performed using GraphPad Prism 10.0. Data are presented as mean ± SD, and normality was assessed using the Shapiro–Wilk test. Escape latency during MWM acquisition training was analyzed using two-way repeated-measures ANOVA followed by Šídák’s multiple-comparisons test. Normally distributed multi-group data were analyzed using one-way ANOVA followed by Dunnett’s or Tukey’s multiple-comparisons test, as appropriate, whereas non-normally distributed data were analyzed using the Kruskal–Wallis test followed by Dunn’s multiple-comparisons test. Test statistics, degrees of freedom, and adjusted *p* values were reported where applicable. *p* < 0.05 was considered statistically significant.

## 3. Results

### 3.1. Behavioral Tests

Representative swimming trajectories in the MWM test are shown in Figure 2A,B. During the place navigation trial, mice in the control and sham groups showed relatively direct swimming paths toward the platform area, whereas Aβ_1–42_-induced AD model mice predominantly swam along the periphery of the maze. Mice in the donepezil- and CBD-treated groups showed more focused trajectories toward the platform area than model mice. In the probe trial, trajectories in the control and sham groups were concentrated in the quadrant where the platform had previously been located. In contrast, AD model mice continued to swim mainly along the maze periphery. Donepezil- and CBD-treated mice showed increased trajectories within the former platform quadrant compared with AD model mice.

Quantitative analysis of MWM probe-trial performance is shown in Figure 2C–E. The number of platform crossings differed significantly among the experimental groups (Kruskal–Wallis test, H (6) = 19.512, *p* = 0.0034). Compared with the control group, Aβ_1–42_-induced AD model mice showed a significantly lower platform crossover number (# *p* < 0.05, Figure 2C). Compared with the model group, the donepezil, CBD-L, CBD-M, and CBD-H groups showed significantly higher platform crossover numbers (* *p* < 0.05 or ** *p* < 0.01, Figure 2C).

Platform residence time also differed significantly among the groups (Kruskal–Wallis test, H (6) = 28.747, *p* < 0.0001). Compared with the control group, the model group showed significantly reduced platform residence time (### *p* < 0.001, Figure 2D). Compared with the model group, the CBD-M and CBD-H groups showed significantly longer platform residence time (** *p* < 0.01 and * *p* < 0.05, respectively), whereas the differences in the donepezil and CBD-L groups did not reach statistical significance (Figure 2D).

Total movement distance differed significantly among the experimental groups (Kruskal–Wallis test, H (6) = 21.505, *p* = 0.0015). Compared with the control group, the model group showed a shorter movement distance (### *p* < 0.001, Figure 2E), indicating impaired spatial memory performance. Compared with the model group, the donepezil, CBD-L, CBD-M, and CBD-H groups showed increased movement distance (* *p* < 0.05 or ** *p* < 0.01, Figure 2E). These results indicate that CBD treatment attenuated Aβ_1–42_-induced deficits in MWM probe-trial performance.

Escape latency during the acquisition phase is shown in Figure 2F. Two-way repeated-measures ANOVA revealed significant effects of treatment group (F (6, 77) = 53.984, *p* < 0.0001) and training day (F (5, 385) = 53.028, *p* < 0.0001), with no significant group × day interaction (F (30, 385) = 1.110, *p* = 0.3189). Šídák’s multiple-comparisons test showed longer escape latencies in the model group than in the control group and shorter latencies in the donepezil- and CBD-treated groups than in the model group on multiple training days (adjusted *p* < 0.05). These results indicate that CBD treatment improved spatial learning performance in Aβ_1–42_-induced mice.

Representative trajectories from the object recognition test are shown in Figure S1. Mice in the control group showed more frequent exploration of the novel object, whereas model mice mainly moved along the periphery of the open field. Compared with the model group, mice in the donepezil- and CBD-treated groups displayed increased exploration of the novel object area.

Quantitative analysis of NOR performance showed a significant overall difference in the discrimination index among the experimental groups (one-way ANOVA, F (6, 72) = 5.898, *p* < 0.0001; Figure 2G). Compared with the control group, the model group showed a significantly lower discrimination index (### *p* < 0.001, Figure 2G), indicating impaired recognition memory. Compared with the model group, the donepezil, CBD-L, CBD-M, and CBD-H groups showed significantly higher discrimination indices (* *p* < 0.05, ** *p* < 0.01, or *** *p* < 0.001, Figure 2G). These results indicate that CBD treatment attenuated Aβ_1–42_-induced impairment in recognition memory.

### 3.2. H&E Staining and Immunofluorescence Analysis

Representative H&E-stained hippocampal sections are shown in Figure 3A. The control and sham groups exhibited relatively preserved hippocampal architecture, with neurons arranged in a comparatively regular manner. In contrast, Aβ_1–42_-induced mice showed apparent disruption of hippocampal cytoarchitecture, irregular neuronal arrangement, and prominent vacuolar and degenerative morphological alterations. The donepezil- and CBD-treated groups exhibited relatively preserved hippocampal morphology and more orderly neuronal organization compared with the model group, with less prominent degenerative changes. As H&E staining was used for qualitative histopathological assessment in the present study, these observations were interpreted as complementary morphological evidence suggesting that CBD treatment was associated with attenuation of Aβ_1–42_-induced hippocampal injury.

Representative hippocampal immunofluorescence images for tau-related staining are shown in Figure 3B, and quantitative analysis of the p-tau/tau ratio is shown in Figure 4A. The p-tau/tau ratio differed significantly among the experimental groups (one-way ANOVA, F (6, 14) = 9.351, *p* = 0.0003; Figure 4A). Compared with the control group, the model group showed a significantly increased p-tau/tau ratio (### *p* < 0.001, Figure 4A), indicating enhanced tau phosphorylation in the hippocampus after Aβ_1–42_ injection. Compared with the model group, the donepezil, CBD-L, CBD-M, and CBD-H groups showed significantly lower p-tau/tau ratios (*** *p* < 0.001, Figure 4A). These results indicate that CBD treatment was associated with reduced tau phosphorylation in the hippocampus of Aβ_1–42_-induced mice.

### 3.3. Effects of CBD on AEA, 2-AG, and FAAH in the Mouse Hippocampus

Hippocampal AEA and 2-AG levels are shown in Figure 4B,C. AEA levels differed significantly among the experimental groups (one-way ANOVA, F (6, 35) = 5.373, *p* = 0.0005; Figure 4B). Compared with the control group, Aβ_1–42_-induced model mice showed a significant reduction in AEA levels (# *p* < 0.05, Figure 4B). Compared with the model group, AEA levels were significantly increased in the donepezil, CBD-L, CBD-M, and CBD-H groups (* *p* < 0.05 or ** *p* < 0.01, Figure 4B). No significant overall difference in 2-AG levels was detected among the experimental groups (one-way ANOVA, F (6, 35) = 0.800, *p* = 0.5768; Figure 4C). These results indicate that CBD treatment was associated with increased hippocampal AEA levels in Aβ_1–42_-induced mice.

FAAH levels in the hippocampus are shown in Figure 4D. FAAH levels differed significantly among the experimental groups (one-way ANOVA, F (6, 47) = 2.693, *p* = 0.0249; Figure 4D). Compared with the control group, the model group showed a significant increase in FAAH levels (# *p* < 0.05, Figure 4D). Compared with the model group, FAAH levels were significantly reduced in the donepezil, CBD-L, CBD-M, and CBD-H groups (* *p* < 0.05 or ** *p* < 0.01, Figure 4D). Together with the changes in AEA, these findings suggest that the CBD associated increase in hippocampal AEA was related to reduced FAAH levels.

### 3.4. Effects of CBD on Synaptophysin, CB1R, and BDNF/TrkB Signaling in the Hippocampus of Aβ_1–42_ Induced Mice

Western blot analysis was performed to assess synaptophysin, CB1R, BDNF, TrkB, and p-TrkB protein abundance in the hippocampus of Aβ_1–42_-induced mice. Representative blots are shown in Figure 5A,B, and quantitative analyses are shown in Figure 5C–F. Synaptophysin abundance differed significantly among the experimental groups (one-way ANOVA, F (6, 14) = 21.024, *p* < 0.0001; Figure 5C). Compared with the control group, the model group showed significantly reduced synaptophysin abundance normalized to GAPDH (## *p* < 0.01, Figure 5C). Compared with the model group, synaptophysin abundance was significantly increased in the donepezil, CBD-L, CBD-M, and CBD-H groups (* *p* < 0.05 or ** *p* < 0.01, Figure 5C).

CB1R/β-actin ratios differed significantly among the experimental groups (one-way ANOVA, F (6, 14) = 4.317, *p* = 0.0114; Figure 5D). BDNF/β-actin ratios also differed significantly among the groups (one-way ANOVA, F (6, 14) = 21.855, *p* < 0.0001; Figure 5E). Similarly, a significant overall difference was observed in the p-TrkB/TrkB ratio (one-way ANOVA, F (6, 14) = 20.632, *p* < 0.0001; Figure 5F). The model group also showed significantly lower CB1R/β-actin, BDNF/β-actin, and p-TrkB/TrkB ratios than the control group (### *p* < 0.001, Figure 5D–F). Compared with the model group, CB1R abundance was significantly increased in the donepezil, CBD-M, and CBD-H groups (* *p* < 0.05 or ** *p* < 0.01, Figure 5D). BDNF/β-actin and p-TrkB/TrkB ratios were significantly increased in the donepezil, CBD-L, CBD-M, and CBD-H groups compared with the model group (** *p* < 0.01 or *** *p* < 0.001, Figure 5E,F). These data indicate that Aβ_1–42_ injection reduced hippocampal synaptic protein abundance and suppressed CB1R- and BDNF/TrkB associated signaling markers, whereas CBD treatment partially reversed these molecular changes.

### 3.5. Effects of Aβ_1–42_ and CBD on PC12 Cell Viability and Morphology

The effects of Aβ_1–42_ and CBD on PC12 cell viability are shown in Figure 6B,C. PC12 cell viability differed significantly among the Aβ_1–42_ concentration groups (one-way ANOVA, F (6, 14) = 17.400, *p* < 0.0001; Figure 6B). Aβ_1–42_ reduced PC12 cell viability in a concentration-dependent manner. Treatment with 0.5 μM Aβ_1–42_ did not significantly alter cell viability, whereas 1–20 μM Aβ_1–42_ significantly reduced viability compared with the untreated control group (*** *p* < 0.001, Figure 6B). Because 1 μM Aβ_1–42_ produced a significant but moderate reduction in viability, this concentration was selected for subsequent model induction.

PC12 cell viability also differed significantly among the CBD concentration groups (one-way ANOVA, F (7, 16) = 60.670, *p* < 0.0001; Figure 6C). CBD exerted a non-linear effect on PC12 cell viability. Compared with the untreated control group, 10 and 15 μM CBD significantly increased cell viability (*** *p* < 0.001 or * *p* < 0.05, Figure 6C), whereas 25 and 30 μM CBD significantly reduced cell viability (*** *p* < 0.001, Figure 6C). No significant changes were observed at 2.5, 5, or 20 μM CBD. Based on these results, 10 μM CBD was selected for subsequent experiments.

Representative light microscopy images of PC12 cells are shown in Figure 6A. Control cells displayed a spindle-like morphology with extended neurite-like processes. Aβ_1–42_-treated model cells showed morphological alterations, including reduced or retracted processes, less distinct cell boundaries, and an increased number of rounded or shrunken cells, as indicated by the arrows. In the Aβ_1–42_ + CBD group, cell morphology was partially preserved, with more visible neurite-like processes and fewer rounded cells than in the model group. These observations suggest that CBD partially attenuated Aβ_1–42_-induced morphological injury in PC12 cells.

### 3.6. Effects of CBD on Synaptophysin, CB1R, and BDNF/TrkB Signaling in Aβ_1–42_ Treated PC12 Cells

Western blot analysis was used to determine synaptophysin, CB1R, BDNF, TrkB, and p-TrkB protein abundance in PC12 cells. The experimental groups were control, Aβ_1–42_ model, CBD alone, Aβ_1–42_ + CBD, and Aβ_1–42_ + CBD + AM251 co-treatment groups, as indicated in Figure 7A,B. Quantitative analyses are shown in Figure 7C–F.

Synaptophysin/GAPDH abundance differed significantly among the experimental groups (one-way ANOVA, F (4, 10) = 10.038, *p* = 0.0016; Figure 7C). Compared with the control group, Aβ_1–42_ treatment significantly reduced synaptophysin/GAPDH abundance (** *p* < 0.01, Figure 7C). Compared with the model group, synaptophysin abundance was significantly increased in the Aβ_1–42_ + CBD group (** *p* < 0.01, Figure 7C). Co-treatment with AM251 significantly reduced synaptophysin abundance compared with the Aβ_1–42_ + CBD group (## *p* < 0.01, Figure 7C).

CB1R/β-actin ratios differed significantly among the experimental groups (one-way ANOVA, F (4, 10) = 25.155, *p* < 0.0001; Figure 7D). Significant overall differences were also observed in BDNF/β-actin (one-way ANOVA, F (4, 10) = 109.235, *p* < 0.0001; Figure 7E) and p-TrkB/TrkB ratios (one-way ANOVA, F (4, 10) = 300.667, *p* < 0.0001; Figure 7F). Aβ_1–42_ treatment also significantly reduced CB1R/β-actin, BDNF/β-actin, and p-TrkB/TrkB ratios compared with the control group (* *p* < 0.05 or *** *p* < 0.001, Figure 7D–F). Compared with the model group, Aβ_1–42_ + CBD treatment significantly increased CB1R, BDNF, and p-TrkB/TrkB levels (** *p* < 0.01 or *** *p* < 0.001, Figure 7D–F). AM251 co-treatment significantly attenuated the CBD-associated increases in CB1R, BDNF, and p-TrkB/TrkB levels compared with the Aβ_1–42_ + CBD group (# *p* < 0.05 or ### *p* < 0.001, Figure 7D–F). These results support the involvement of CB1R in CBD-associated modulation of BDNF/TrkB signaling in Aβ_1–42_-treated PC12 cells.

## 4. Discussion

### 4.1. CBD Ameliorates Cognitive Impairment and Hippocampal Injury in Aβ_1–42_-Induced Mice

Aβ accumulation is widely considered a key pathological event in AD and is closely associated with tau hyperphosphorylation, neuroinflammation, oxidative stress, synaptic dysfunction, and neuronal injury, which collectively contribute to disease progression [19,30,31,32,33,34]. In the present study, behavioral assessments showed that Aβ_1–42_-induced mice exhibited deficits in spatial learning, spatial memory, and recognition memory, whereas CBD treatment significantly improved these behavioral outcomes. In parallel, Aβ_1–42_ induced marked hippocampal histopathological alterations and an increased p-tau/tau ratio, whereas CBD attenuated these pathological changes and reduced tau hyperphosphorylation. These findings indicate that the improvement in cognitive performance following CBD treatment was accompanied by attenuation of Aβ_1–42_-associated hippocampal injury.

Synaptophysin is commonly used as a presynaptic marker and reflects synaptic integrity [35]. Increasing evidence indicates that preservation of synaptic structure and function is closely associated with cognitive resilience in AD. Experimental studies have shown that restoration of synaptic proteins, dendritic spine density, and synaptic number can occur in parallel with improvements in learning and memory performance in AD-related models [36,37]. Consistent with these observations, reduced hippocampal synaptophysin abundance in Aβ_1–42_-induced mice was accompanied by impaired MWM and NOR performance, whereas CBD treatment partially restored synaptophysin abundance together with improved behavioral performance. These parallel molecular and behavioral changes suggest that preservation of synaptic integrity may contribute to the cognitive improvement associated with CBD treatment.

### 4.2. CBD Modulates the AEA–FAAH–CB1R Axis and BDNF/TrkB Signaling

The ECS has been implicated in AD-related pathological processes [38,39,40,41,42]. In the present study, Aβ_1–42_ injection reduced hippocampal AEA levels and increased FAAH levels, whereas 2-AG levels were not significantly altered. CBD treatment increased AEA levels while reducing FAAH levels, suggesting that the CBD-associated elevation of AEA may be related to reduced FAAH-mediated degradation. Notably, the absence of significant changes in 2-AG may reflect the distinct metabolic regulation of the two major endocannabinoids. AEA and 2-AG are regulated by partially distinct metabolic pathways, with FAAH serving as a major catabolic enzyme for AEA, whereas 2-AG is predominantly hydrolyzed by monoacylglycerol lipase (MAGL). Thus, the selective changes in AEA and FAAH observed in the present study suggest that CBD may preferentially modulate the AEA–FAAH branch of the ECS under the present experimental conditions.

BDNF/TrkB signaling is closely associated with neuronal survival, synaptic plasticity, and cognitive function [43,44,45]. Previous studies have suggested functional interactions between endocannabinoid signaling and the BDNF/TrkB pathway [46,47]. In the present study, Aβ_1–42_-induced reductions in hippocampal CB1R, BDNF, and p-TrkB/TrkB levels were partially reversed by CBD treatment. Together with the CBD-associated increase in AEA and reduction in FAAH, these findings support a working hypothesis in which modulation of the AEA–FAAH system is associated with restoration of CB1R-related signaling and recovery of BDNF/TrkB signaling under Aβ_1–42_-induced injury. Restoration of this signaling environment may, in turn, contribute to preservation of synaptic integrity and improvement of cognitive function. Thus, the coordinated changes in AEA, FAAH, CB1R, and BDNF/TrkB-related markers provide a plausible mechanistic framework linking the molecular effects of CBD to its observed neuroprotective and cognitive effects.

### 4.3. CB1R Contributes to CBD-Associated Regulation of BDNF/TrkB Signaling in Aβ_1–42_- Treated PC12 Cells

The in vitro PC12 cell experiments provided additional functional evidence for the involvement of CB1R in CBD-associated regulation of BDNF/TrkB signaling. Aβ_1–42_ exposure reduced cell viability and induced morphological injury, accompanied by reductions in synaptophysin, CB1R, BDNF, and the p-TrkB/TrkB ratio. CBD treatment partially reversed these alterations. Importantly, pharmacological blockade of CB1R with AM251 attenuated several CBD-associated molecular effects, including restoration of synaptophysin, CB1R, BDNF, and p-TrkB/TrkB levels. These findings support a functional contribution of CB1R to CBD-associated regulation of BDNF/TrkB-related signaling under Aβ_1–42_-induced injury conditions and provide additional support for the proposed linkage between CB1R-related signaling and the BDNF/TrkB pathway.

### 4.4. Differential and Non-Linear Responses to CBD Across Doses

The effects of CBD were not strictly monotonic across the tested doses or biological endpoints. For example, although all three CBD doses significantly increased the p-TrkB/TrkB ratio relative to the model group, the magnitude of this response did not increase proportionally with dose. Importantly, the statistical annotations in Figure 5F represent comparisons with the model group and do not demonstrate that CBD-L was statistically superior to CBD-M or CBD-H. Therefore, the numerically higher p-TrkB/TrkB ratio observed in the CBD-L group should not be interpreted as evidence that the low dose was universally more effective than the medium or high doses.

A non-linear concentration response was also observed in the PC12 cell viability assay. CBD at 10 and 15 μM significantly increased cell viability, whereas higher concentrations of 25 and 30 μM reduced viability. These findings indicate that increasing CBD exposure does not necessarily result in a proportional enhancement of a given biological response and that different molecular or cellular endpoints may exhibit different sensitivities to CBD. Such differences may arise from pathway saturation, compensatory regulation, or the engagement of additional CBD-sensitive targets as exposure increases. However, these possibilities were not directly tested in the present study and should therefore be regarded as potential explanations rather than established mechanisms. Overall, the present findings support beneficial effects of CBD across the tested in vivo dose range but do not indicate a simple linear dose–response relationship.

### 4.5. Polypharmacological Nature of CBD and Broader Mechanistic Implications

Collectively, the present study suggests that CBD attenuates Aβ_1–42_-induced cognitive impairment, hippocampal injury, tau hyperphosphorylation, and synaptic protein loss. These protective effects were accompanied by increased hippocampal AEA levels, reduced FAAH levels, and restoration of CB1R and BDNF/TrkB-associated signaling, suggesting that modulation of the AEA–FAAH–CB1R axis and its functional association with BDNF/TrkB signaling may contribute, at least in part, to the neuroprotective effects of CBD (Figure 8).

However, CBD is a pharmacologically pleiotropic compound whose biological actions are not restricted to CB1R-related signaling. CBD has been reported to interact with or modulate multiple molecular targets, including CB2 receptors, serotonin 5-HT1A receptors, TRPV1 channels, and PPAR-γ [48,49]. These targets are involved in diverse biological processes relevant to AD, including synaptic regulation, neuroinflammation, oxidative stress, and neuronal survival. These additional CBD-sensitive targets were not directly assessed in the present study. Therefore, the behavioral and molecular improvements observed in the present study may reflect the coordinated actions of CBD across multiple signaling systems. Our findings specifically support the potential involvement of the AEA–FAAH–CB1R axis and its association with BDNF/TrkB signaling under the present experimental conditions, but this pathway should be regarded as one potentially important component of CBD-mediated neuroprotection rather than as its exclusive mechanism of action.

### 4.6. Limitations and Future Perspectives

This study has several limitations. First, only relatively young male mice were used, and the intracerebroventricular Aβ_1–42_ model does not fully reproduce the aging-related, progressive, and multifactorial features of human AD. Because both aging and sex may influence synaptic function, neuroinflammatory responses, and endocannabinoid signaling, the present findings cannot be directly generalized to aged animals or females. Future studies incorporating aged animals, both sexes, and/or transgenic AD models are therefore needed to further establish the generalizability of these findings.

Second, the present study focused primarily on the AEA–FAAH–CB1R axis and BDNF/TrkB signaling. Key enzymes involved in endocannabinoid synthesis and metabolism were not assessed, including N-acyl phosphatidylethanolamine-specific phospholipase D (NAPE-PLD), which contributes to AEA biosynthesis, and diacylglycerol lipase-α (DAGL-α) and MAGL, which are involved in 2-AG synthesis and degradation, respectively. Therefore, although hippocampal 2-AG levels were not significantly altered, possible changes in 2-AG metabolic turnover cannot be excluded. Future studies incorporating a more comprehensive assessment of ECS metabolic enzymes and endocannabinoid dynamics are warranted.

Finally, CB1R antagonism was examined only in Aβ_1–42_-treated PC12 cells and was not performed in vivo. Thus, although the AM251 experiments support a functional contribution of CB1R to CBD-associated regulation of BDNF/TrkB signaling in vitro, the causal requirement for CB1R in the behavioral and neuropathological effects of CBD remains to be established in animals. Further in vivo pharmacological blockade or genetic manipulation of CB1R will be needed to establish the causal contribution of CB1R to the effects of CBD.

## 5. Conclusions

In summary, CBD ameliorated Aβ_1–42_-induced cognitive impairment and hippocampal injury, accompanied by reduced tau hyperphosphorylation and partial restoration of synaptic integrity. Based on the integrated in vivo and in vitro findings, the CBD-associated reduction in FAAH and elevation of AEA were associated with restoration of CB1R-related signaling and recovery of BDNF/TrkB signaling under Aβ_1–42_-induced injury. The attenuation of several CBD-associated molecular effects by AM251 further supports a functional contribution of CB1R to this signaling network in vitro. Thus, modulation of the AEA–FAAH–CB1R axis and its functional association with BDNF/TrkB signaling may represent one plausible mechanism contributing to the neuroprotective effects of CBD.

## Figures and Tables

**Figure 1:**
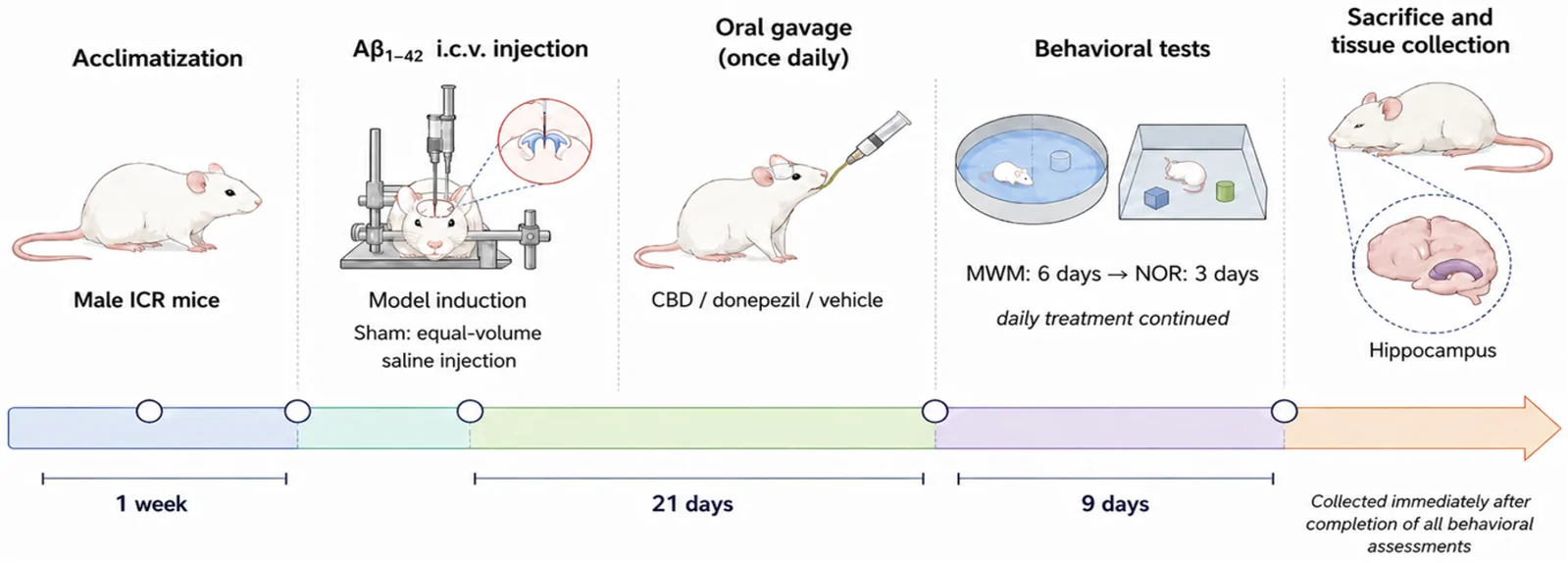
Schematic representation of the experimental design and timeline.

**Figure 2:**
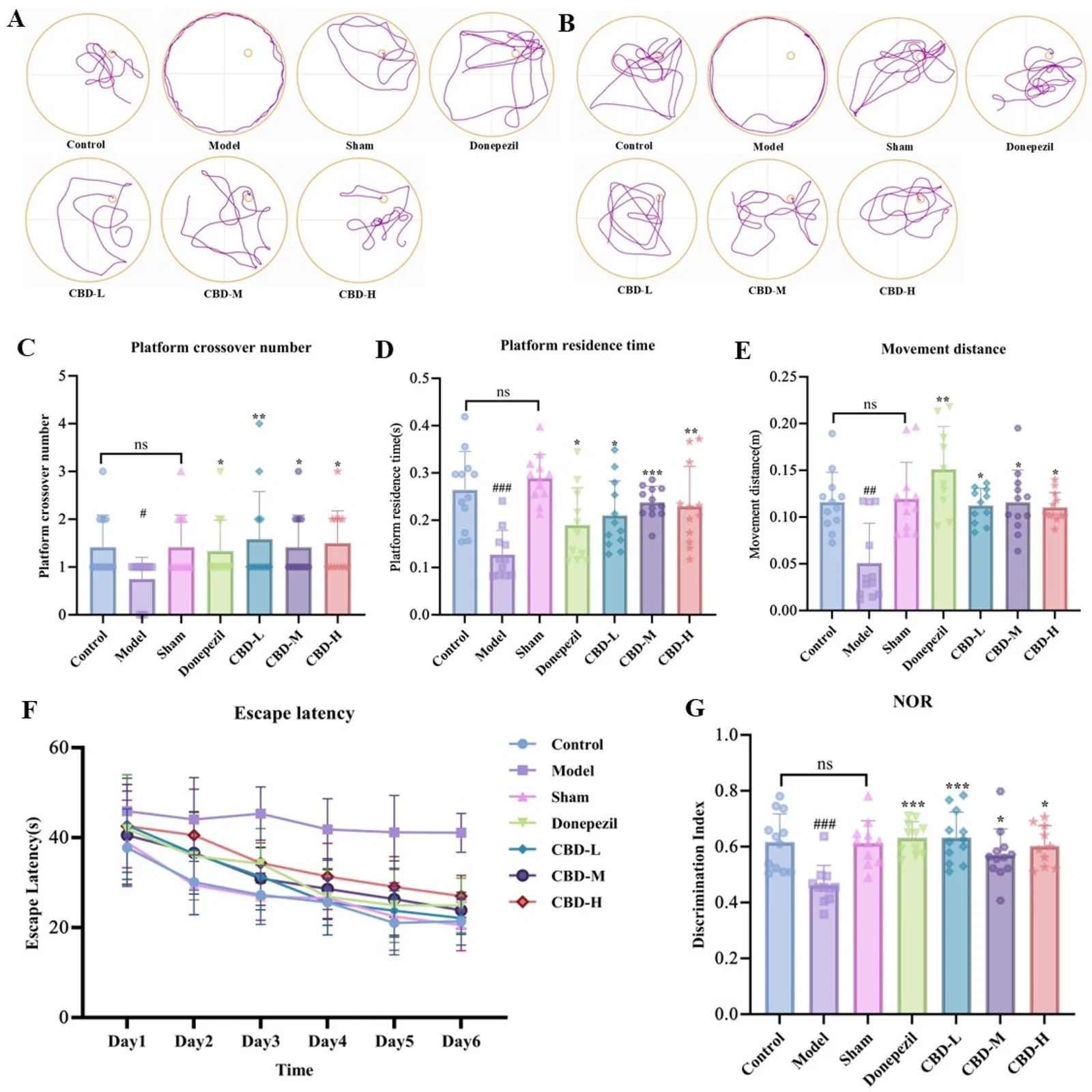
CBD improved spatial learning, memory retrieval, and object recognition in Aβ_1–42_ induced AD model mice. (**A**) Representative swimming paths during Morris water maze (MWM) acquisition training. (**B**) Representative swimming paths during the probe trial. (**C**) Number of platform crossings during the probe trial. (**D**) Time spent in the target platform zone. (**E**) Total movement distance during the probe trial. (**F**) Escape latency during MWM acquisition training from day 1 to day 6. (**G**) Discrimination index in the NOR. Data are presented as mean ± SD, *n* = 10–12 mice per group. For (**C**–**E**), statistical comparisons were performed using the Kruskal–Wallis test followed by Dunn’s multiple-comparisons test. For (**F**), two-way repeated-measures ANOVA followed by Šídák’s multiple-comparisons test was used. For (**G**), one-way ANOVA followed by Tukey’s multiple-comparisons test was used. ns, not significant. # *p* < 0.05, ## *p* < 0.01, ### *p* < 0.001 vs. Control, * *p* < 0.05, ** *p* < 0.01, *** *p* < 0.001 vs. Model.

**Figure 3:**
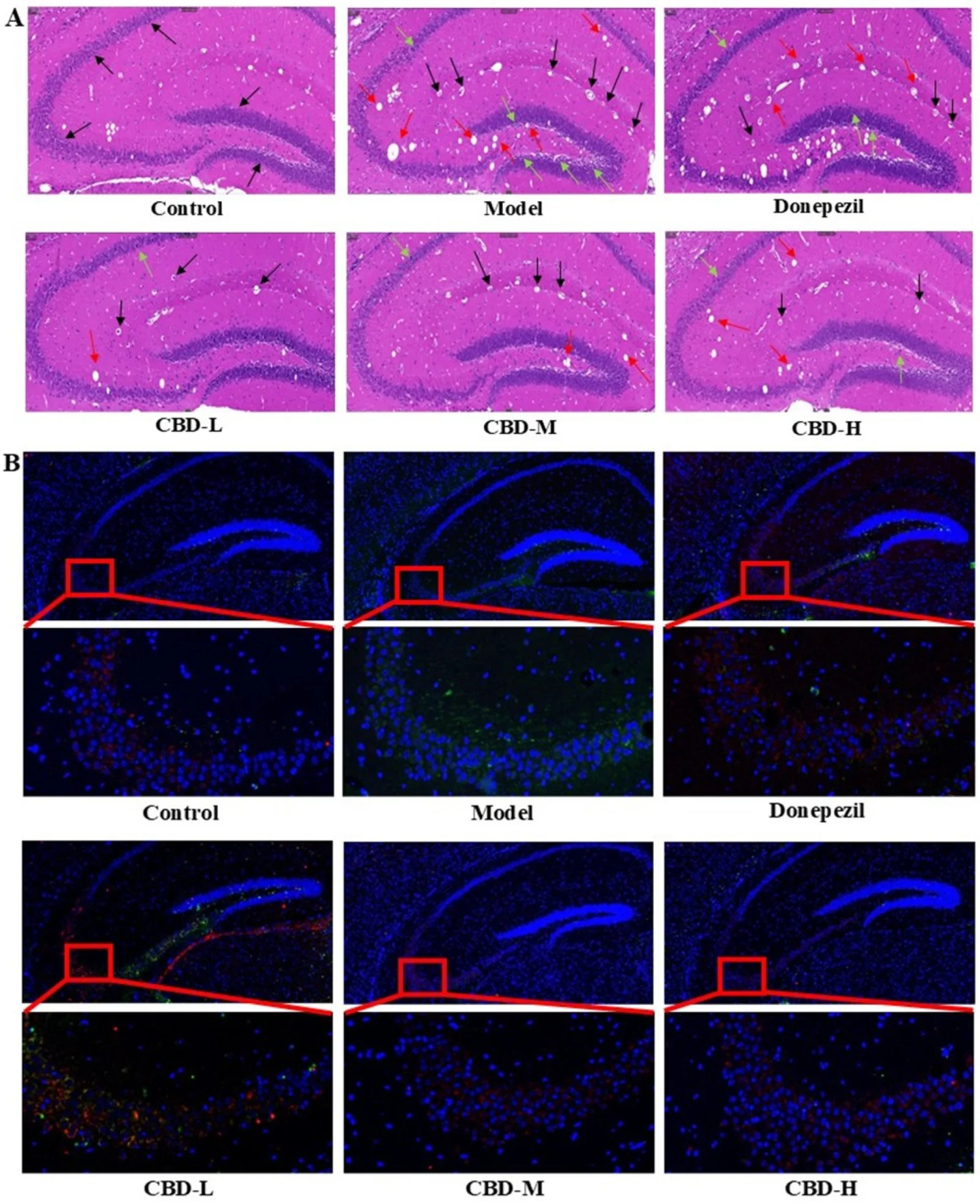
CBD alleviated hippocampal histopathological alterations and tau related pathology in Aβ_1–42_-induced AD model mice. (**A**) Representative hematoxylin and eosin (H&E) stained images of the hippocampus at 20× magnification. Arrows indicate representative histopathological alterations. (**B**) Representative immunofluorescence images showing tau and phosphorylated tau (p-tau) staining in the hippocampus. Low-magnification images were acquired at 5× magnification, and the enlarged images show the boxed regions at 20× magnification. Blue indicates DAPI-stained nuclei, red indicates tau, and green indicates p-tau.

**Figure 4:**
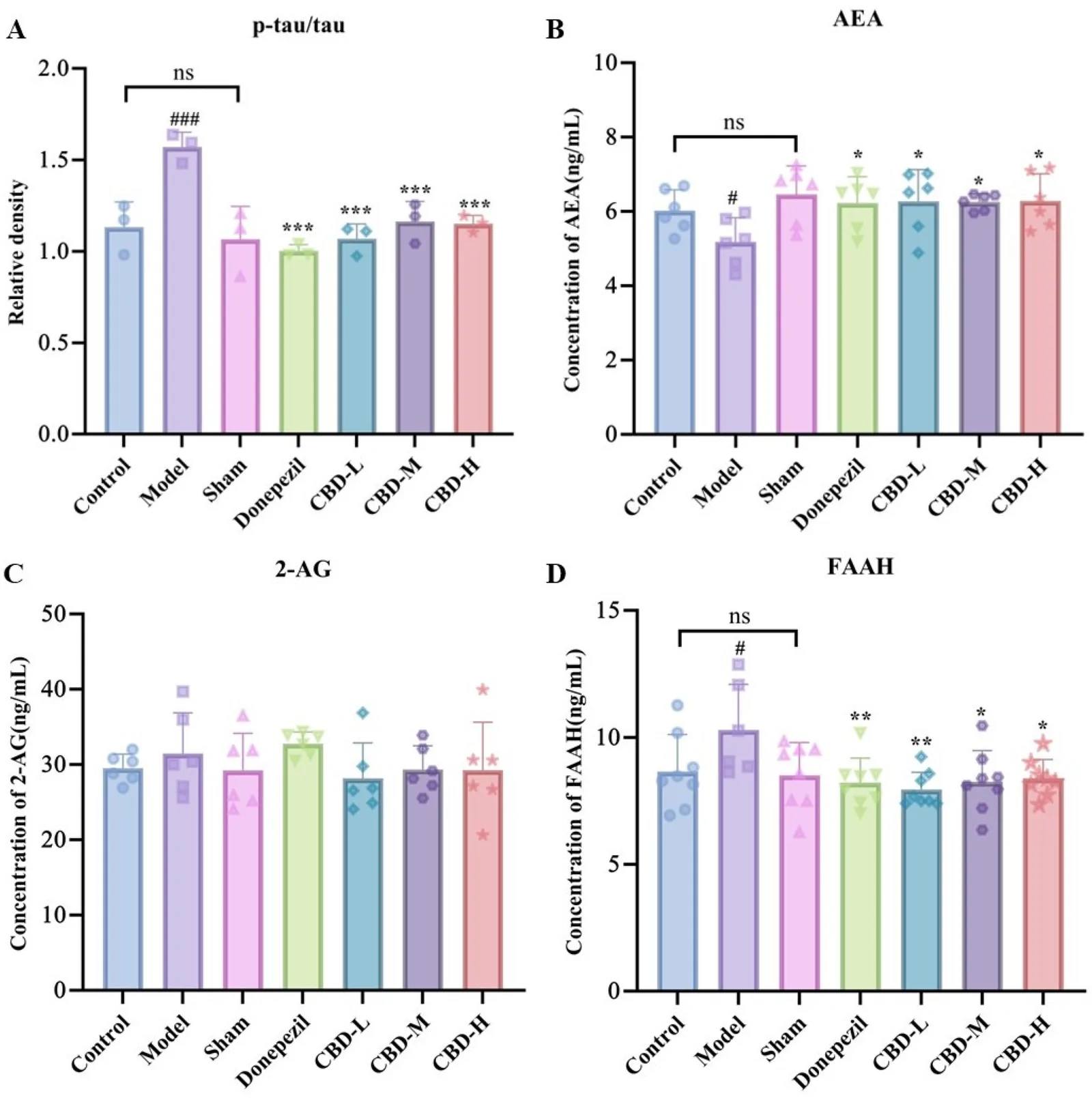
CBD reduced tau phosphorylation and modulated hippocampal ECS related markers in Aβ_1–42_-induced AD model mice. (**A**) Quantification of the p-tau/tau fluorescence intensity ratio in the hippocampus. (**B**) Hippocampal anandamide (AEA) concentration. (**C**) Hippocampal 2-arachidonoylglycerol (2-AG) concentration. (**D**) Hippocampal fatty acid amide hydrolase (FAAH) concentration. Data are presented as mean ± SD, *n* = 3 biological replicates per group for (**A**), *n* = 6 biological replicates per group for (**B**,**C**), and *n* = 6–8 biological replicates per group for (**D**). Statistical comparisons were performed using one-way ANOVA followed by Tukey’s multiple-comparisons test. ns, not significant. # *p* < 0.05, ### *p* < 0.001 vs. Control, * *p* < 0.05, ** *p* < 0.01, *** *p* < 0.001 vs. Model.

**Figure 5:**
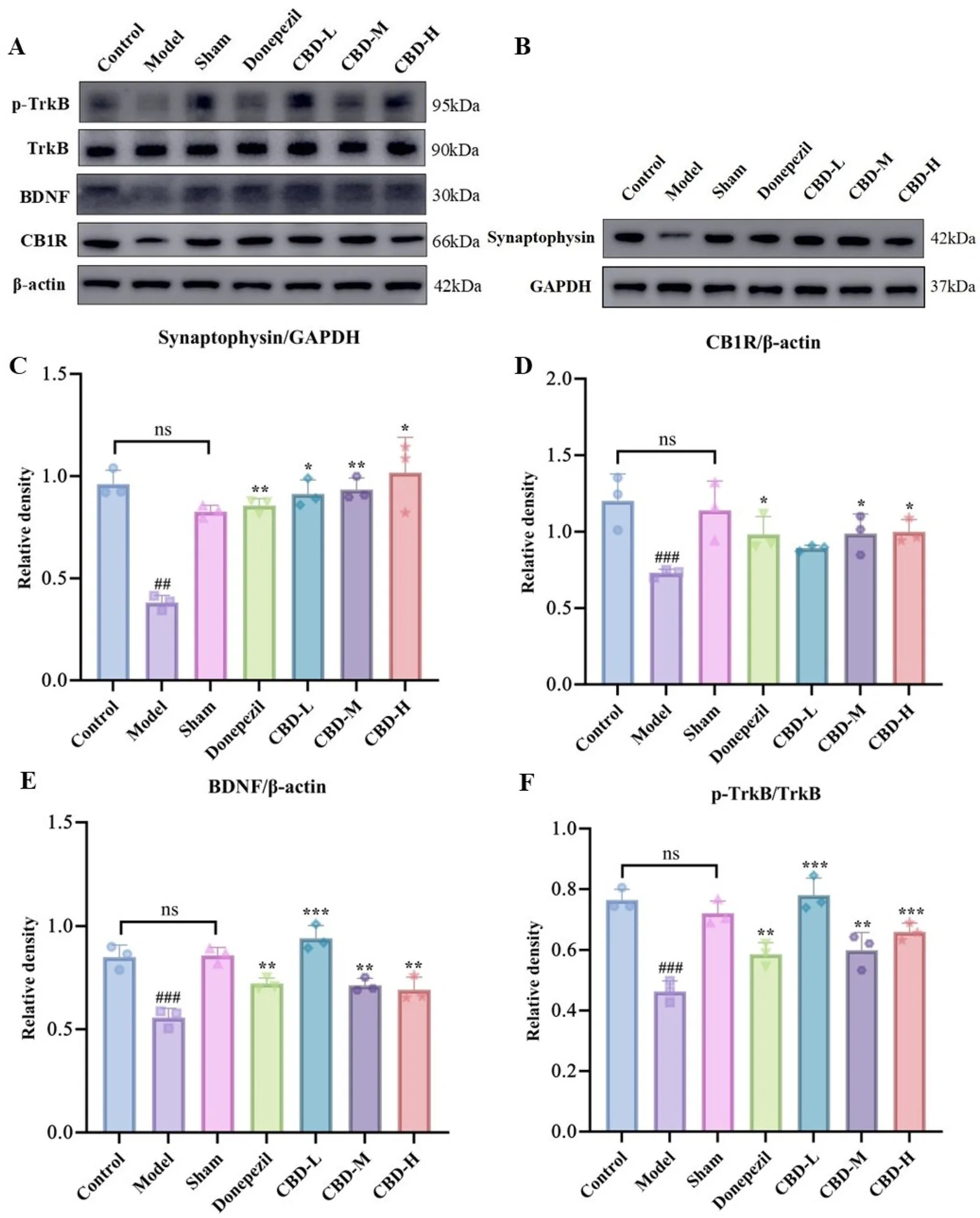
CBD restored hippocampal synaptic protein expression and CB1R–BDNF/TrkB signaling in Aβ_1–42_-induced AD model mice. (**A**) Representative Western blots of p-TrkB, total TrkB, BDNF, CB1R, and β-actin. (**B**) Representative Western blots of synaptophysin and GAPDH. Molecular masses are indicated on the right. (**C**) Densitometric analysis of synaptophysin normalized to GAPDH. (**D**) Densitometric analysis of CB1R normalized to β-actin. (**E**) Densitometric analysis of BDNF normalized to β-actin. (**F**) Densitometric analysis of p-TrkB normalized to total TrkB. Data are presented as mean ± SD; *n* = 3 biological replicates per group. Statistical comparisons were performed using one-way ANOVA followed by Tukey’s multiple-comparison test. ns, not significant. ## *p* < 0.01, ### *p* < 0.001 vs. Control, * *p* < 0.05, ** *p* < 0.01, *** *p* < 0.001 vs. Model. The original Western blot images can be found in Supplementary File S1.

**Figure 6:**
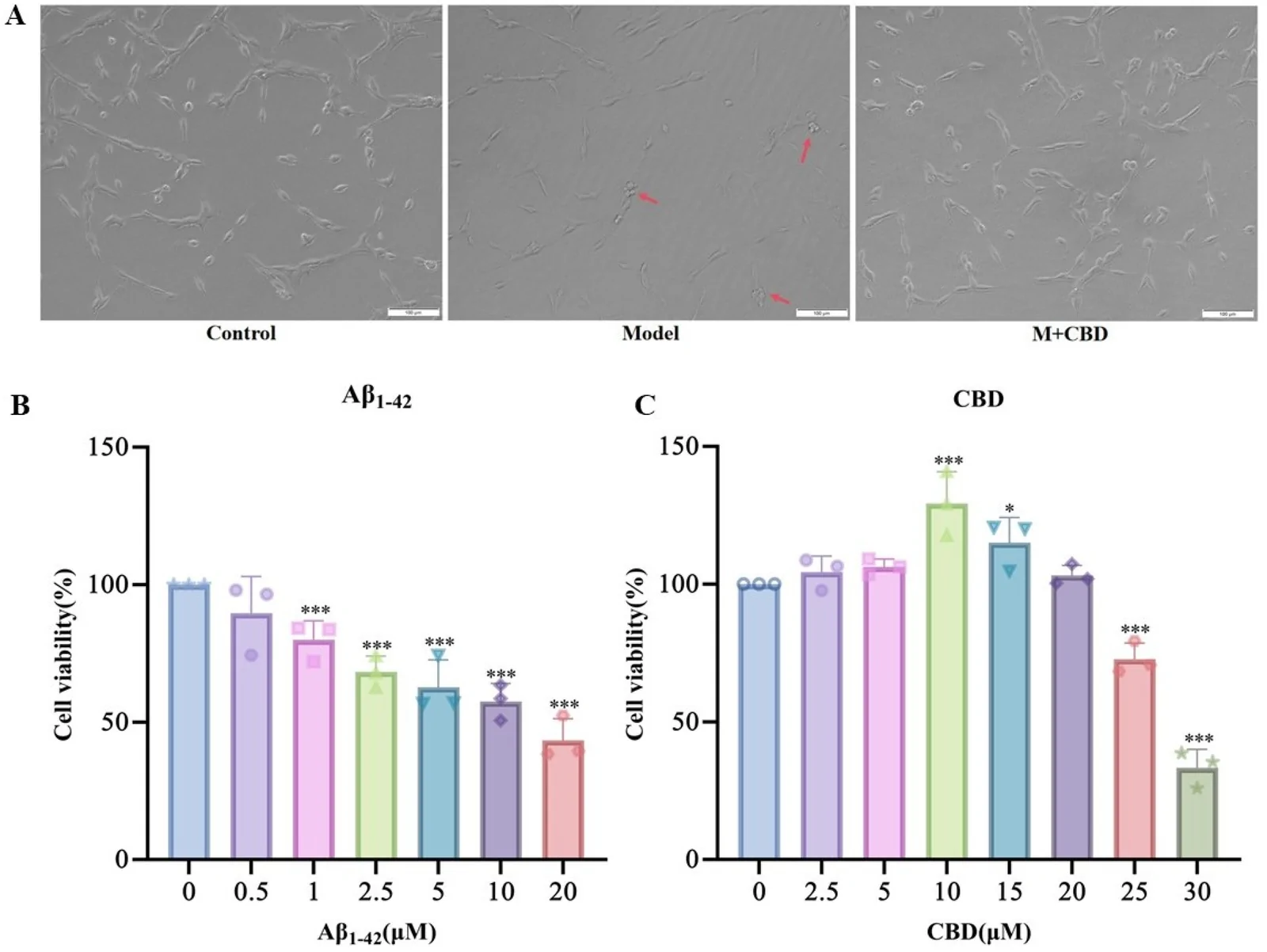
Establishment of the Aβ_1–42_-treated PC12 cell injury model and selection of CBD treatment concentrations. (**A**) Representative phase-contrast images of PC12 cells in the Control, Model, and Aβ_1–42_ + CBD groups. Arrows indicate representative injured or morphologically altered cells. Scale bars, 100 μm. (**B**) Effects of increasing Aβ_1–42_ concentrations on PC12 cell viability. (**C**) Effects of increasing CBD concentrations on PC12 cell viability. Cell viability was assessed using the assay described in the Methods. Data are presented as mean ± SD, *n* = 3 independent experiments. Statistical comparisons for (**B**,**C**) were performed using one-way ANOVA followed by Dunnett’s multiple-comparisons test, with the 0 μM group as the reference control. * *p* < 0.05, *** *p* < 0.001 vs. the 0 μM control.

**Figure 7:**
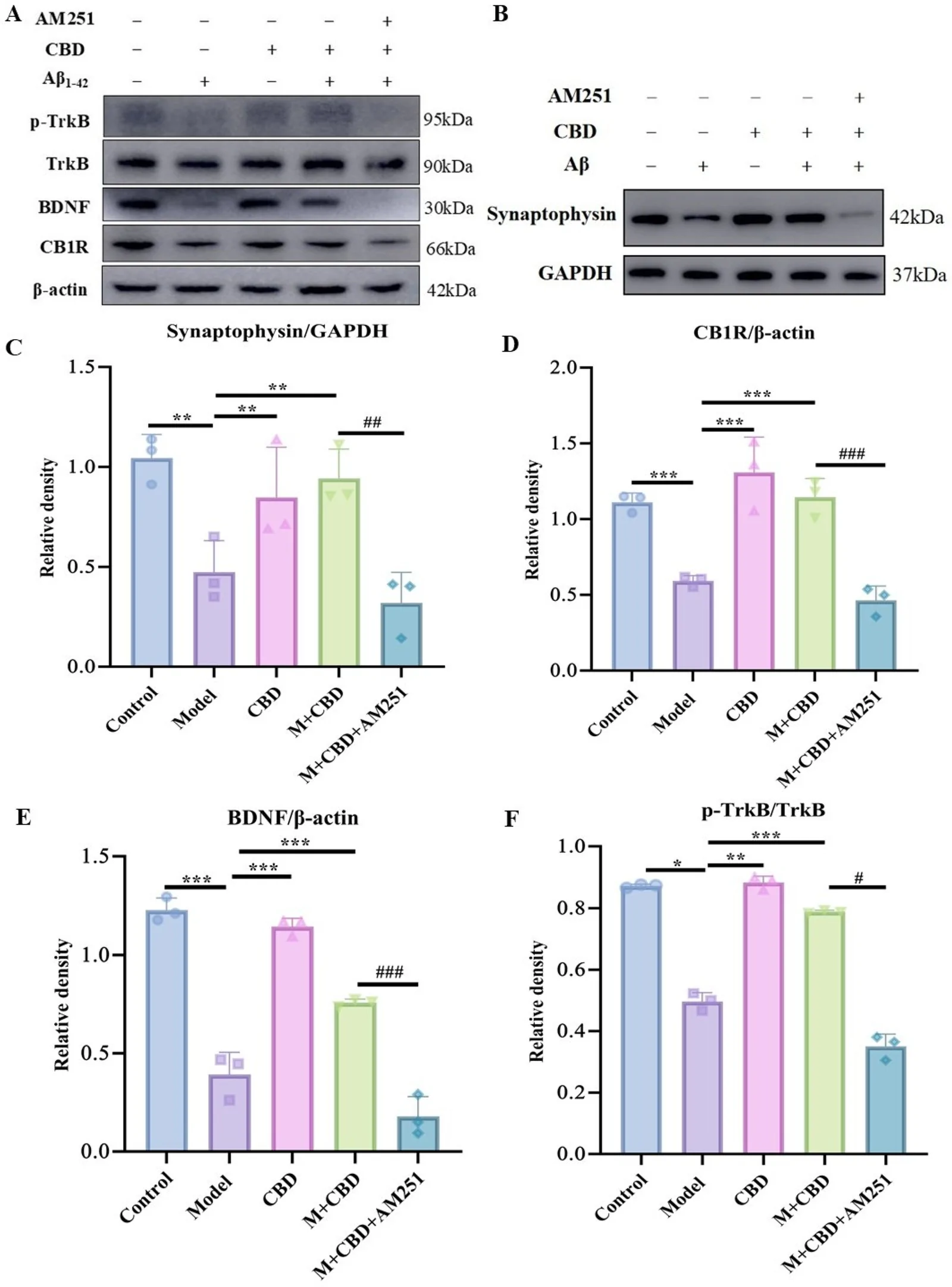
AM251 attenuated CBD-mediated restoration of synaptic protein expression and BDNF/TrkB signaling in Aβ_1–42_-treated PC12 cells. (**A**) Representative Western blots of p-TrkB, total TrkB, BDNF, CB1R, and β-actin. (**B**) Representative Western blots of synaptophysin and GAPDH. The treatment matrix indicates the presence (+) or absence (−) of AM251, CBD, and Aβ_1–42_. (**C**) Densitometric analysis of synaptophysin normalized to GAPDH. (**D**) Densitometric analysis of CB1R normalized to β-actin. (**E**) Densitometric analysis of BDNF normalized to β-actin. (**F**) Densitometric analysis of p-TrkB normalized to total TrkB. Data are presented as mean ± SD; *n* = 3 biological replicates per group. Statistical comparisons were performed using one-way ANOVA followed by Tukey’s multiple-comparisons test. * symbols indicate significant differences for the bracketed comparisons; # symbols indicate significant differences vs. the Aβ_1–42_ + CBD group. * *p* < 0.05, ** *p* < 0.01, *** *p* < 0.001; # *p* < 0.05, ## *p* < 0.01, ### *p* < 0.001. The original Western blot images can be found in Supplementary File S1.

**Figure 8:**
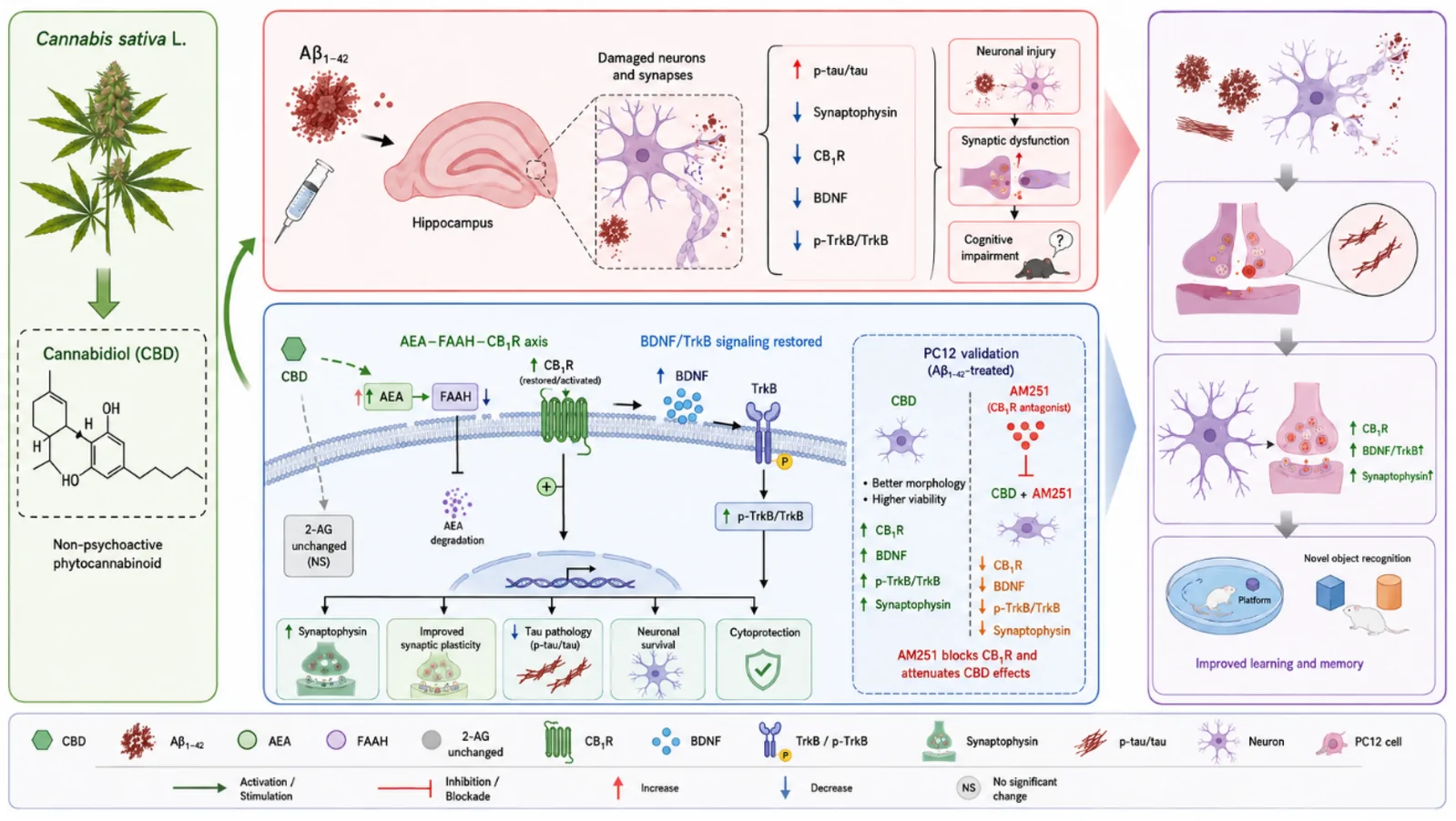
Schematic illustration of the proposed mechanism associated with the effects of CBD on Aβ_1–42_-induced cognitive impairment and neuronal injury. Based on the present findings, CBD-associated modulation of the AEA–FAAH–CB1R axis and BDNF/TrkB signaling may contribute to reduced tau hyperphosphorylation, preservation of synaptic integrity, and improvement of cognitive function.

## Data Availability

Supporting data for the conclusions of this study are available from the corresponding author upon request.

## Supplementary Materials

The following supporting information can be downloaded at: https://www.mdpi.com/article/10.3390/biom16101473/s1, Figure S1: Representative tracking trajectories in the novel object recognition (NOR) test; Supplementary File S1: Original Western blot images corresponding to Figure 5 and Figure 7.
