# Supplementary material for: Cannabidiol Alleviates Aβ1–42-Induced Cognitive Deficits and Neuronal Injury Associated with Modulation of the AEA–FAAH–CB1R Axis and BDNF/TrkB Signaling
Source: Biomolecules. 2026 Oct 10;16(10):1473. doi: 10.3390/biom16101473 (PMC13702573; doi:10.3390/biom16101473)
Supplement: Supplementary file 1 [file biomolecules-16-01473-s001.zip › biomolecules-4496767-Figure S1.pdf]

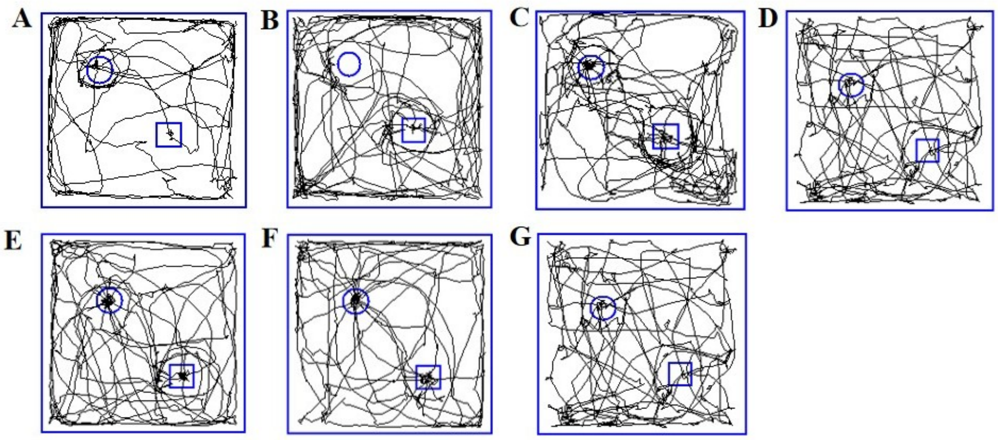

Figure S1. Representative tracking trajectories in the novel object recognition (NOR) test.

A, Control group; B, Model group; C, Sham group; D, Donepezil group; E, CBD-L group; F, CBD-M group; G, CBD-H group.
