# Supplementary material for: Cannabidiol Alleviates Aβ1–42-Induced Cognitive Deficits and Neuronal Injury Associated with Modulation of the AEA–FAAH–CB1R Axis and BDNF/TrkB Signaling
Source: Biomolecules. 2026 Oct 10;16(10):1473. doi: 10.3390/biom16101473 (PMC13702573; doi:10.3390/biom16101473)
Supplement: Supplementary file 1 [file biomolecules-16-01473-s001.zip › biomolecules-4496767-Supplementary-File S1.pdf]

Fig.5 (A)(B)

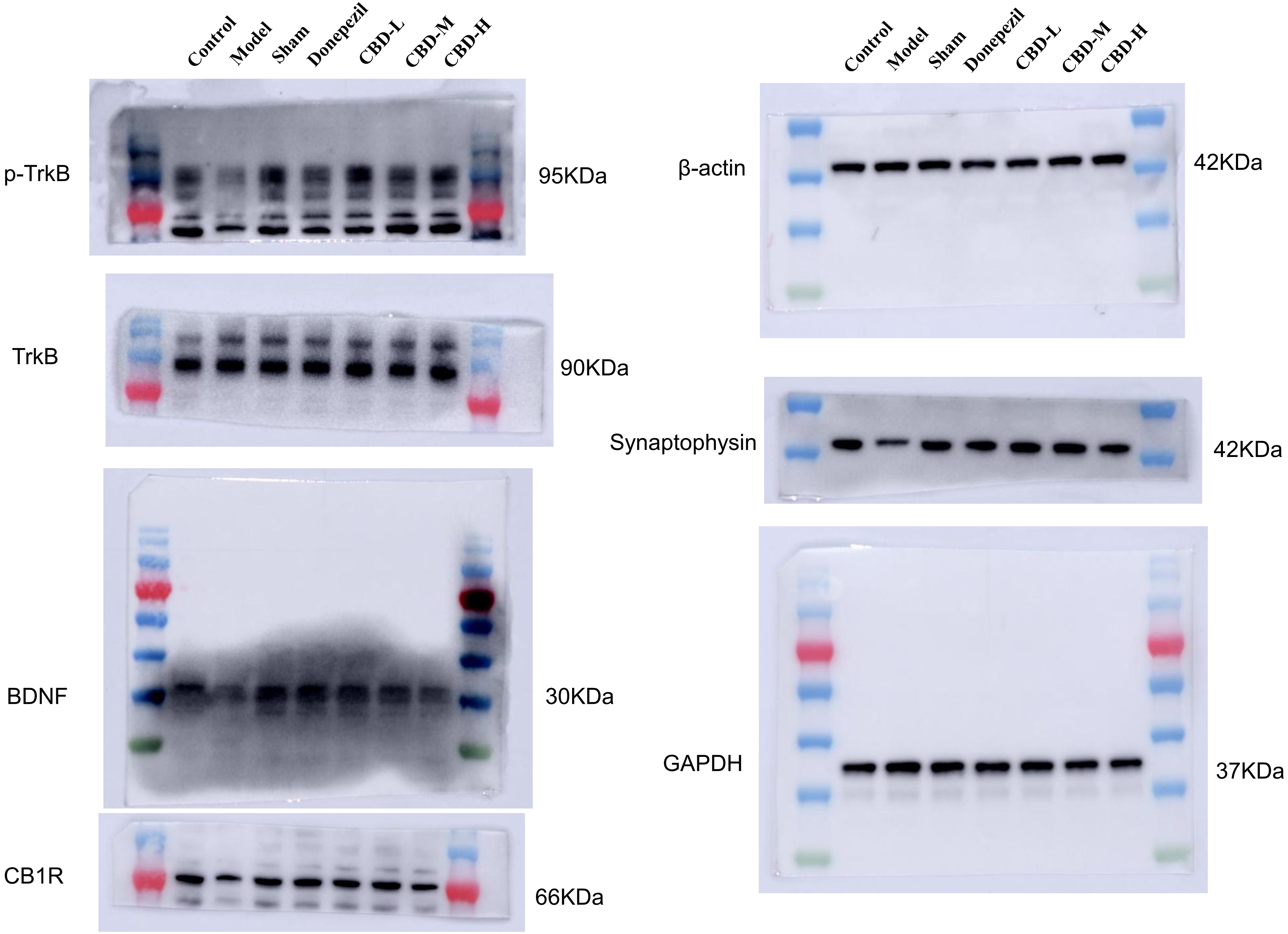
Control
Model
Sham
Donepezil
CBD-L
CBD-M
CBD-H
p-TrkB
95KDa
TrkB
90KDa
BDNF
30KDa
CB1R
66KDa
β-actin
42KDa
Synaptophysin
42KDa
GAPDH
37KDa

Fig.7 (A)(B)

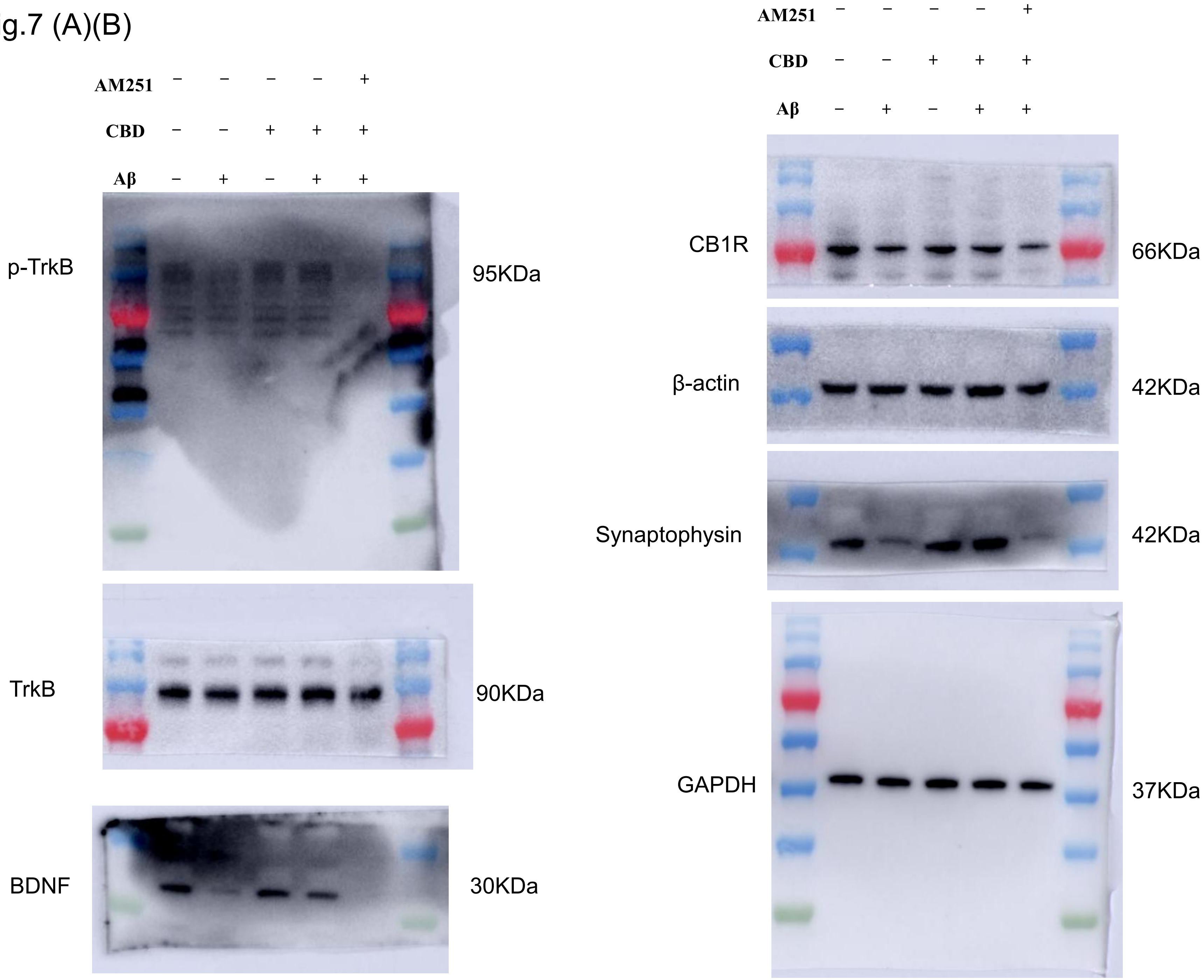
AM251 − − − − +
CBD − − + + +
Aβ − + − + +
p-TrkB
95KDa
TrkB
90KDa
BDNF
30KDa
AM251 − − − − +
CBD − − + + +
Aβ − + − + +
CB1R
66KDa
β-actin
42KDa
Synaptophysin
42KDa
GAPDH
37KDa

Original uncropped Western blot images corresponding to Figure 5.

p-TrkB

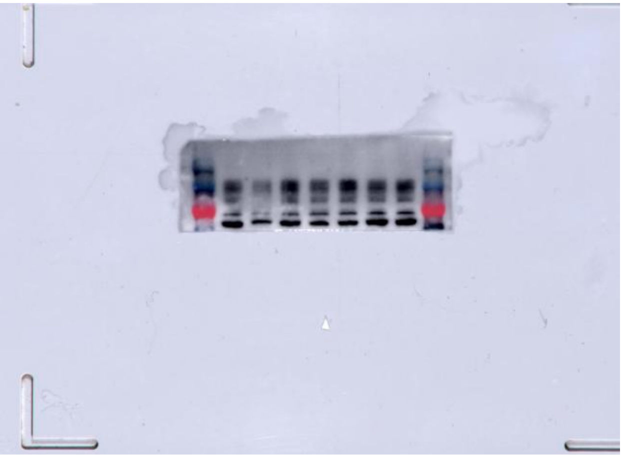

95KDa

BDNF

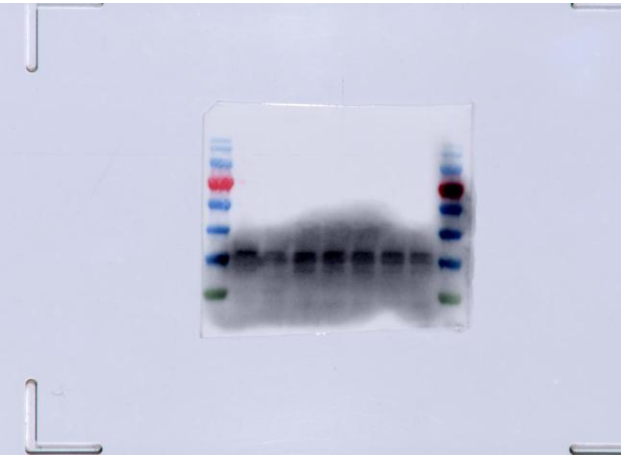

30KDa

TrkB

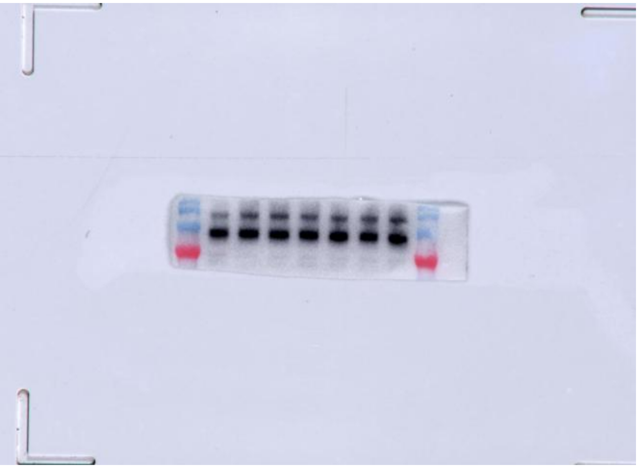

90KDa

CB1R

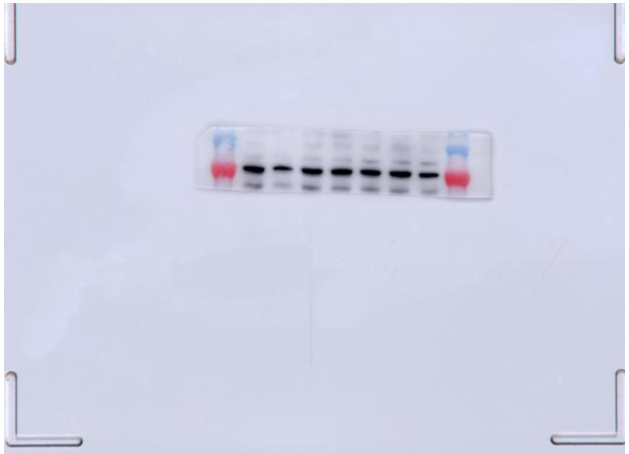

66KDa

β-actin

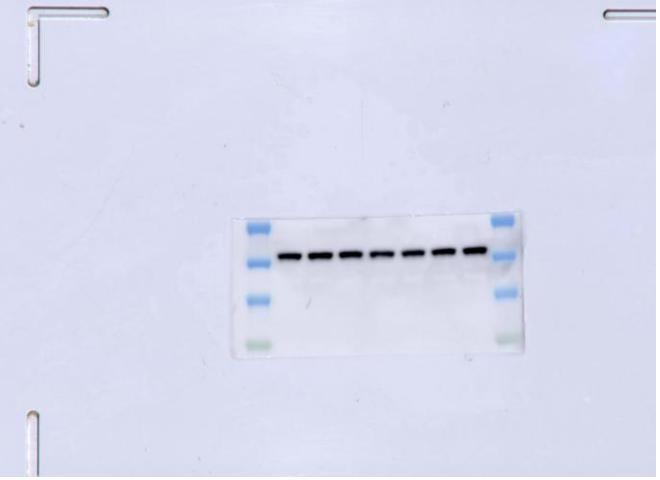

42KDa

Synaptophysin

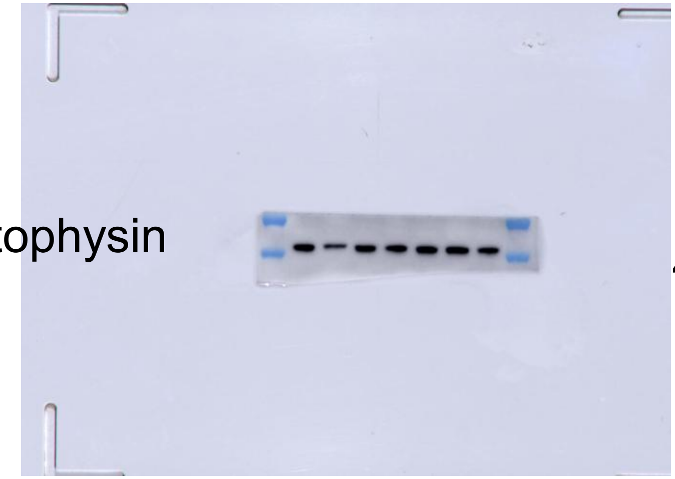

42KDa

GAPDH

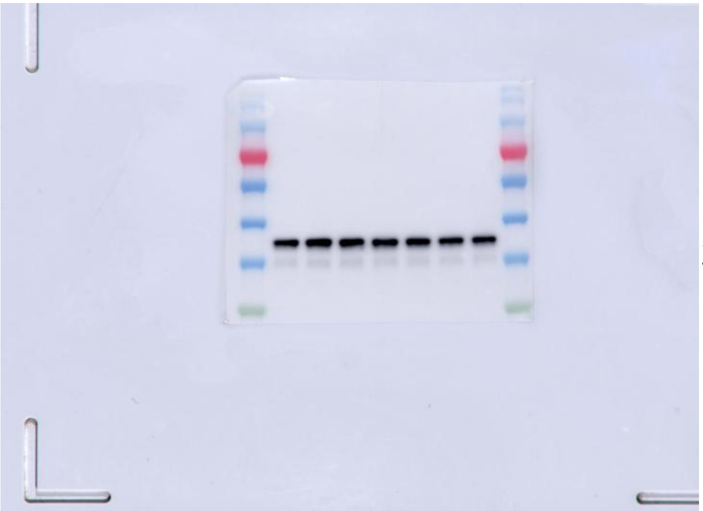

37KDa

Original uncropped Western blot images corresponding to Figure 7.

p-TrkB

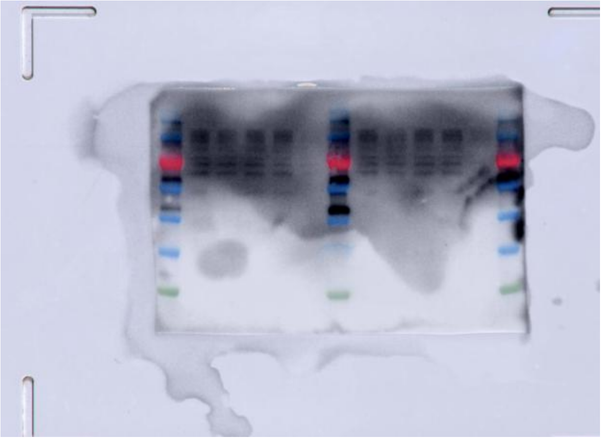

95KDa

BDNF

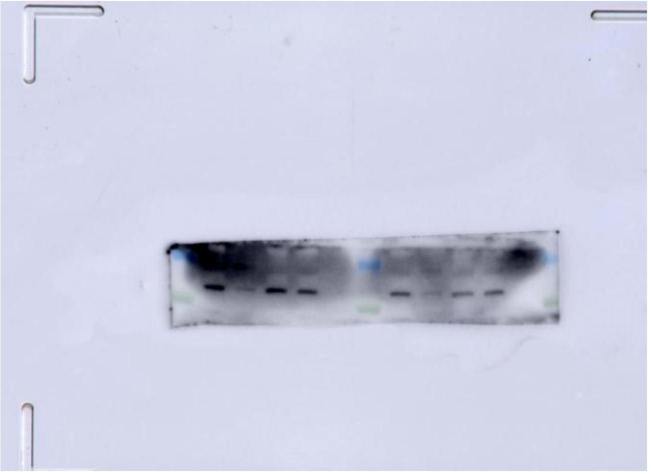

30KDa

TrkB

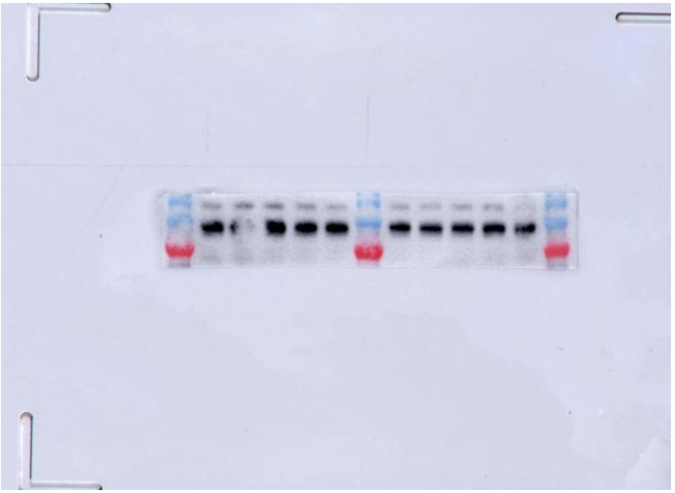

90KDa

CB1R

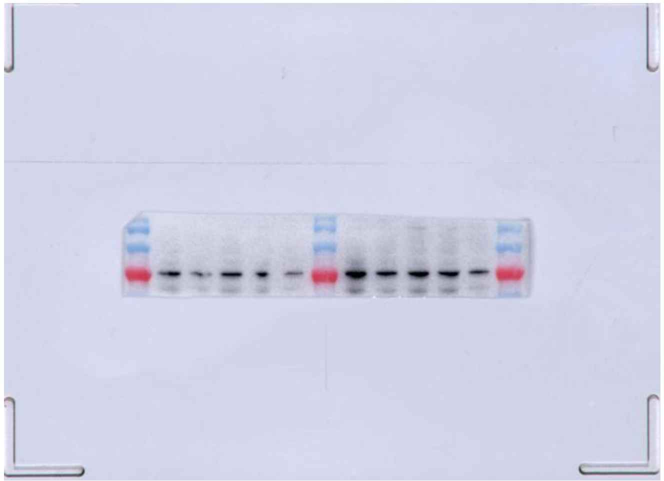

66KDa

β-actin

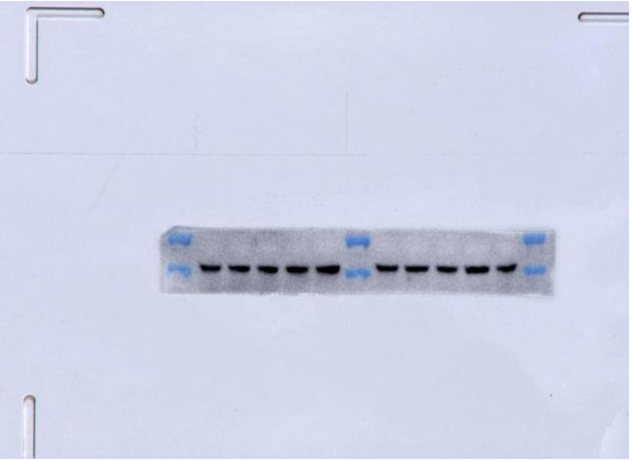

42KDa

GAPDH

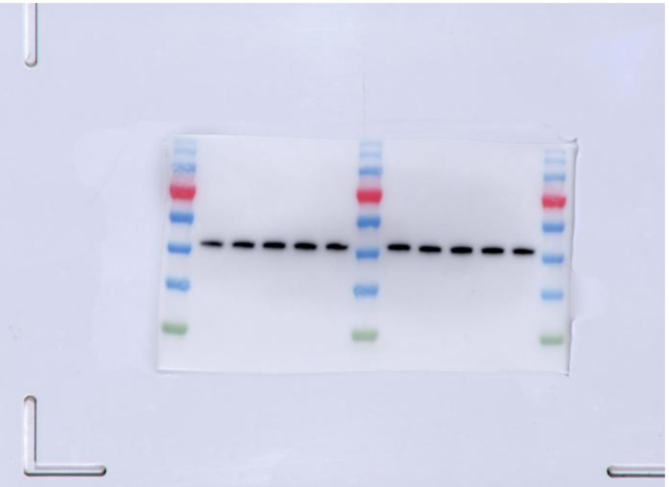

37KDa

Synaptophysin

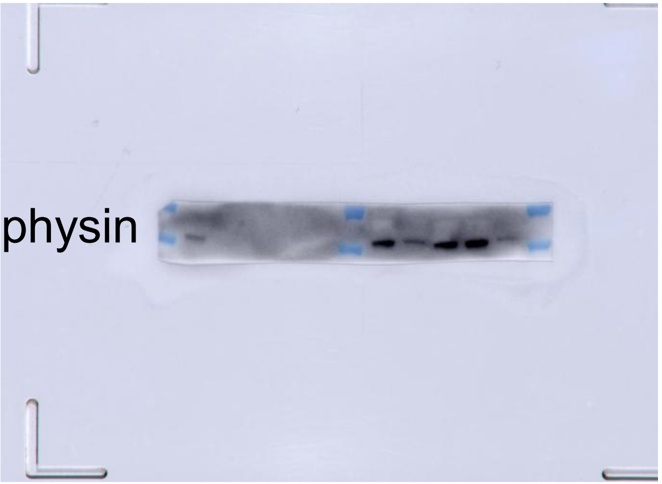

42KDa
